# Activation of β-Catenin Signaling in CD133-Positive Dermal Papilla Cells Drives Postnatal Hair Growth

**DOI:** 10.1371/journal.pone.0160425

**Published:** 2016-07-29

**Authors:** Linli Zhou, Mingang Xu, Yongguang Yang, Kun Yang, Randall R. Wickett, Thomas Andl, Sarah E. Millar, Yuhang Zhang

**Affiliations:** 1 Division of Pharmaceutical Sciences, College of Pharmacy, University of Cincinnati, Cincinnati, Ohio 45267, United States of America; 2 Department of Cancer Biology, Vontz Center for Molecular Studies, University of Cincinnati College of Medicine, Cincinnati, Ohio 45267, United States of America; 3 Burnett School of Biomedical Sciences, University of Central Florida, Orlando, Florida 32826, United States of America; 4 Department of Dermatology, University of Pennsylvania Perelman School of Medicine, Philadelphia, Pennsylvania 19104, United States of America; Simon Fraser University, CANADA

## Abstract

The hair follicle dermal papilla (DP) contains a unique prominin-1/CD133-positive (CD133+) cell subpopulation, which has been shown to possess hair follicle-inducing capability. By assaying for endogenous CD133 expression and performing lineage tracing using *CD133-CreER*^*T2*^; *ZsGreen1* reporter mice, we find that CD133 is expressed in a subpopulation of DP cells during the growth phase of the murine hair cycle (anagen), but is absent at anagen onset. However, how CD133+ DP cells interact with keratinocytes to induce hair regenerative growth remains unclear. Wnt/β-catenin has long been recognized as a major signaling pathway required for hair follicle morphogenesis, development, and regeneration. Nuclear Wnt/β-catenin activity is observed in the DP during the hair growth phase. Here we show that induced expression of a stabilized form of β-catenin in CD133+ DP cells significantly accelerates spontaneous and depilation-induced hair growth. However, hair follicle regression is not affected in these mutants. Further analysis indicates that CD133+ DP-expressed β-catenin increases proliferation and differentiation of epithelial matrix keratinocytes. Upregulated Wnt/β-catenin activity in CD133+ DP cells also increases the number of proliferating DP cells in each anagen follicle. Our data demonstrate that β-catenin signaling potentiates the capability of CD133+ DP cells to promote postnatal hair growth.

## Introduction

The mature hair follicle undergoes a repetitive process of reconstitution comprising cyclical periods of growth (anagen), regression (catagen) and rest (telogen) [1]. This cycle is maintained throughout the lifespan of an individual by the regenerative behavior of hair follicle epithelial stem cells (HFSCs), which are localized at the lower permanent portion of the hair follicle, known as the bulge [2–4]. It is widely accepted that the mesenchymal component of the hair follicle, the dermal papilla (DP), dictates HFSCs and their progeny to initiate hair growth [5]. Furthermore, a recent report showed that DP cell number specifies the size and shape of murine pelage hairs and their cycling properties [6].

Prominin-1/CD133 has been used as an unique surface stem/progenitor cell marker for varied tissues, including skin [7–9], intestine [10], and a range of tumors, including hepatocellular carcinoma [11], brain tumors [12], and melanoma [13]. In human skin, CD133 expression is detected in a subset of invaginating hair placode cells during early morphogenesis [9]. In mice, a subpopulation of DP cells expresses CD133 during the early anagen stages of the postnatal hair growth cycle [7]. CD133+ DP cells isolated from embryonic or adult DPs have the ability to induce new hair follicles *in vivo*, while CD133- DP cells lack this property [7]. Further studies using an *in vitro* three-dimensional hydrogel culture system and skin reconstitution assay showed that CD133+ DP cells contributed to the establishment of the DP in both primary and secondary hair follicles [14]. However, it remains unclear how CD133+ DP cells interact with epithelial keratinocytes to rebuild the hair follicle structure during the anagen phase.

The role of Wnt/β-catenin signaling in the epithelial compartment has been well established [15]. However, the role of Wnt/β-catenin signaling in the DP for hair follicle induction and growth has remained controversial. β-catenin is localized to the nuclei of dermal condensate cells before the initiation of follicle morphogenesis and is present in DP cells during postnatal anagen [16]. Ablation of β-catenin in Corin+ DP cells causes reduced proliferation of matrix keratinocytes, resulting in early catagen and blockage of entry into a new hair growth cycle [17]. Surprisingly, however, when a stabilized form of β-catenin (*Ctnnb1*^*(Ex3)fl/+*^) was overexpressed in Corin+ DP cells, no change in postnatal hair cycling was observed [18]. Similarly, expression of the same stabilized β-catenin protein in CD133+ DP cells did not result in any phenotype in postnatal hair growth except for an increase in the DP size [19]. It is unclear whether the lack of hair growth phenotypes in these models was due to low activity of the endogenous β-catenin promoter in DP cells.

To address this conundrum, we generated an alternative, tri-transgenic mouse model to activate canonical Wnt signaling in CD133+ DP cells. By contrast with previous gain of function studies, but in line with the known requirements for β-catenin in the DP, we find that expression of ΔN-β-catenin in CD133+ DP cells accelerates postnatal hair growth. Our data suggest manipulation of Wnt signaling in CD133+ DP cells as a potential means to treat hair growth disorders.

## Materials and Methods

### Mice

*CD133-CreER*^*T2*^ (*Prom1*^*C-L*^) mice were generated as described previously [10]. *Rosa-rtTA* (Jax 005670) and *Rosa-CAG-LSL-ZsGreen1* (*ZsGreen1*, Jax 007906) mice were obtained from the Jackson laboratory (Bar Harbor, ME). *tetO-Ctnnb1*^ΔN^ mice were generated by cloning a cDNA fragment that encodes β-catenin protein that lacks 147 amino acids at the N-terminal (ΔN) into a *tetO* promoter vector in Dr. Sarah Millar’s laboratory and will be published in details elsewhere. For lineage tracing, *CD133-CreER*^*T2*^ mice were crossed with *ZsGreen1* mice to generate *CD133-CreER*^*T2*^*; ZsGreen1* reporter mice. To conditionally express ΔN-β-catenin in CD133+ DP cells, *CD133-CreER*^*T2*^; *Rosa-rtTA; tetO-Ctnnb1*^ΔN^ triple transgenic mice were generated by crossing *tetO-Ctnnb1*^ΔN^ mice with *CD133-CreER*^*T2*^ and *Rosa-rtTA* mice for several generations.

Mice were genotyped by polymerase chain reaction (PCR) analysis of genomic DNA extracted from tail biopsies. The presence of *CreER*^*T2*^ transgene in *CD133* locus was genotyped using forward primer: CAGGCTGTTAGCTTGGGTTC and reverse primer 1: AGGCAAATTTTGGTGTACGG. CD133 wild-type allele was genotyped using forward primer with reverse primer 2: TAGCGTGGTCATGAAGCAAC. *ZsGreen1* was genotyped by PCR using the following primer pairs: 1) forward primer: AACCAGAAGTGGCACCTGAC and reverse primer: GGCATTAAAGCAGCGTATCC for mutant allele and 2) forward primer: AAGGGAGCTGCAGTGGAGTA and reverse primer: CCGAAAATCTGTGGGAAGTC for wild type allele. *Rosa-rtTA* was genotyped by PCR using forward primer: AAGTTCATCTGCACCACCG and reverse primer: TCCTTGAAGAAGATGGTGCG. ΔN-β-catenin transgene was genotyped by PCR using forward primer: CCTTGTATCACCATGGACCCTCAT and reverse primer: TAGTGGGATGAGCAGCGTCAAACT. The PCR protocol used was 94°C for 3 min followed by 35 cycles of 94°C for 30 seconds, 62°C for 30 seconds, and 72°C for 40 seconds and a final extension at 72°C for 10 minutes.

### Animal husbandry, diet and transgene induction

All mice were housed in the Laboratory Animal Services Facility of the University of Cincinnati under an artificial 12/12 light-dark cycle and were allowed free access to standard mouse chow and water. All animals were cared daily, seven days per week, by three full-time veterinarians, six veterinary technicians plus animal care staff. The Institutional Animal Care and Use Committee of the University of Cincinnati approved all experimental procedures involving mice.

To induce Cre activity, tamoxifen (TAM) (Sigma-Aldrich, St. Louis, MO) in corn oil (10 mg/ml) was administered to adult mice by intraperitoneal injection at 1mg/g body weight for 7 consecutive days as described in the text. To induce ΔN-β-catenin transgene expression, adult mice at a given age were placed on chow containing 6g/kg doxycycline (Bio-Serv, Laurel, MD, USA). For each time point, at least three to five mutants with control littermates were analyzed.

### Skin biopsies

Stages of normal hair cycle were determined according to the classification system published previously (1). For depilation experiments, 7 to 8-week-old mice were anesthetized with isoflurane, and hairs in a 2 cm^2^ area of mid-dorsal skin were manually plucked with forceps to induce synchronized hair cycling. Hair follicles of mice entering telogen were confirmed by pink to white color change of the skin.

Dorsal skin biopsies were taken from euthanized mice by CO_2_ inhalation, the standard American Veterinary Medical Association method. Death was confirmed by the lack of heartbeat. Cervical dislocation was performed to make sure mice would not recover. Two hours before sacrifice, all mice were given one intraperitoneal injection of 5-bromo-2'-deoxyuridine (BrdU) based on their body weight (50 μg/g of body weight). Hairs on the back of mice were carefully shaved using an electric clipper before harvesting skin biopsies. Collected skin tissues were then processed for paraffin and frozen sectioning.

### Histology and Immunostaining

Paraffin or frozen skin samples were prepared for BrdU incorporation assays and immunostaining according to published protocols. Briefly, paraffin sections were deparaffinized, rehydrated and then performed antigen retrieval in citrate buffer (pH 6.0) using the microwave heating method. Frozen sections were fixed with -20°C acetone for 10 minutes before immunostaining. After washing with PBS, sections were blocked in 10% bovine serum albumin (BSA) in PBS, and subsequently incubated at 4°C overnight with each primary antibody. Next, the slides were washed again with PBS for two times, incubated with the corresponding biotin-conjugated secondary antibodies (Vector lab, Burlingame, CA) at room temperature for 1 hour, and followed by incubating with either flurochrome-labeled Streptavidin for immunofluorescence or VECTASTAIN Elite ABC Reagents (Vector lab, Burlingame, CA) for immunohistochemistry. The slides were examined and images were taken using a Nikon Eclipse 80i fluorescence microscope.

The following primary antibodies were used: anti-CD133 (eBioscience, San Diego, CA, 1:50), anti-β-catenin (Invitrogen, Carlsbad, CA, clone 15B8, 1:1000), anti-Brdu (Abcam, Cambridge, MA, cone BU1/75, 1:25), anti-Ki67 (Imgenex, Littleton, CO, 1:50), anti-cyclin D1 (Cell Signaling, 1:50), anti-LEF1 (Cell Signaling, Danvers, MA, 1:100), anti-Versican (Millipore, Billerica, MA, 1:200), anti-Sox9 (Millipore, Billerica, MA, 1:100), anti-GATA3 (Santa Cruz, Dallas, Texas, 1:50), anti-AE13 (1:25), anti-AE15 (1:25). AE13 and AE15 antibodies were kind gifts from Dr. Tung-Tien Sun (New York University Medical School, New York, NY). Stained slides were examined under a Nikon Eclipse 80i fluorescence microscope and images were analyzed using Adobe Photoshop.

### Lineage tracing

*CD133-CreER*^*T2*^*; ZsGreen1* reporter mice were given daily intraperitoneal injections of tamoxifen from P19 to P25. Skin tissue was then collected at different time points. At each give time point as described in the text, skin samples were taken from the mid-dorsal region, embedded in tissue embedding medium (O.C.T) and serial sectioned for the detection of green fluorescent cells. Frozen sections were co-stained for the expression of alkaline phosphatase in the DP. At least three mice were analyzed for each time point.

### Hair follicle number counting

Stages of hair follicles were determined according to the classification system published previously (1), which gave detailed morphological features for recognizing key stages of murine hair follicle growth (anagen), regression (catagen) and quiescence (telogen) [20]. For each mouse, three pieces of H&E-stained skin biopsies were examined and counted for hair follicle numbers. A minimum of three *CD133-CreER*^*T2*^; *Rosa-rtTA; tetO-Ctnnb1*^ΔN^ mutant mice and three control littermates were counted for each time point.

### DP cell number counting

For each time point, skin biopsies from 3 pairs of *CD133-CreER*^*T2*^; *Rosa-rtTA; tetO-Ctnnb1*^ΔN^ and control mice were counted and compared. Number of DP cells in each hair follicle was identified by counting individual DAPI-stained nucleus within the boundary of the DP (6). Lef1-stained paraffin skin tissues were used to count Lef1+ DP cells in the DP of each hair follicle. Versican-stained paraffin skin tissues were used to count versican+ DP cells in the DP of each hair follicle. For each piece of skin tissue, at least 20 hair follicles are randomly picked for counting.

### Statistical analysis

All graphs were generated using Microsoft Excel (2016). Statistical analysis of difference was carried out by Student’s t-test using a GraphPad Prism 5.01 software package (GraphPad Software Inc., San Diego, CA) and represented as mean ± SEM, with P < 0.05 considered statistically significant.

## Result

### Nuclear β-catenin expression is detected in hair follicle dermal papilla cells

**Fig 1:**
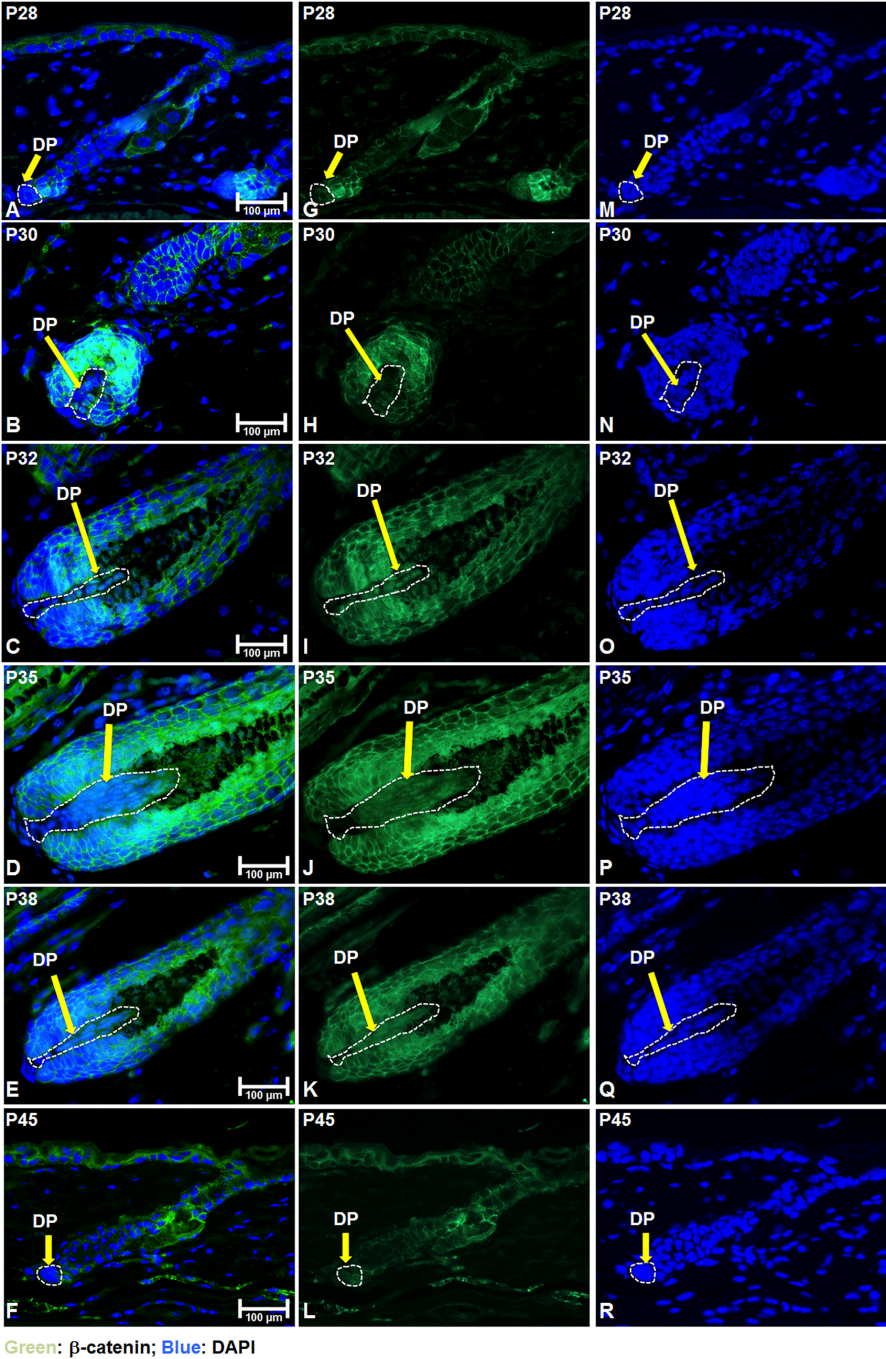
Nuclear β-catenin is detected in the DP during anagen. Skin biopsies of normal C57BL/6 mice were collected at the indicated age and processed for paraffin sections. (**A-F**) Expression of β-catenin (green color) was visualized by immunofluorescence staining and counterstained with DAPI (blue nuclei). The DP was circled by white dashed lines in each hair follicle. (**J-L**) Same β-catenin immunostaining as in (A-F) (green color) without DAPI counterstaining. (**M-R**) DAPI staining of skin tissue sections used in (A-F) (blue nuclei). Scale bar: 100 μm

To assay for β-catenin activity in the DP of postnatal hair follicles, we examined the expression of β-catenin by immunofluorescent staining in postnatal mouse skin. No nuclear β-catenin expression could be detected in DP cells at postnatal day 28 (P28) (Fig 1A, 1G and 1M). From P30 onwards until P38, nuclear expression of β-catenin could be readily observed in DP cells (circled by white dashed lines in Fig 1B–1E and 1H–1K). At each time point, many hair matrix keratinocytes surrounding the DP exhibited membrane localization of β-catenin. When hair follicles enter the regression stage, nuclear β-catenin could no longer be identified in DP cells (Fig 1F and 1L). We have also analyzed β-catenin expression by immunohistochemistry to confirm that nuclear localization of β-catenin in the DP detected by immunofluorescence staining was not an artifact caused by the specific staining protocol (S1A–S1F Fig). These data suggest that activation of Wnt/β-catenin signaling in DP cells in adult hair growth *in vivo* is restricted to mid- to late anagen.

### Specific expression of CD133 in the DP during postnatal hair cycle

**Fig 2:**
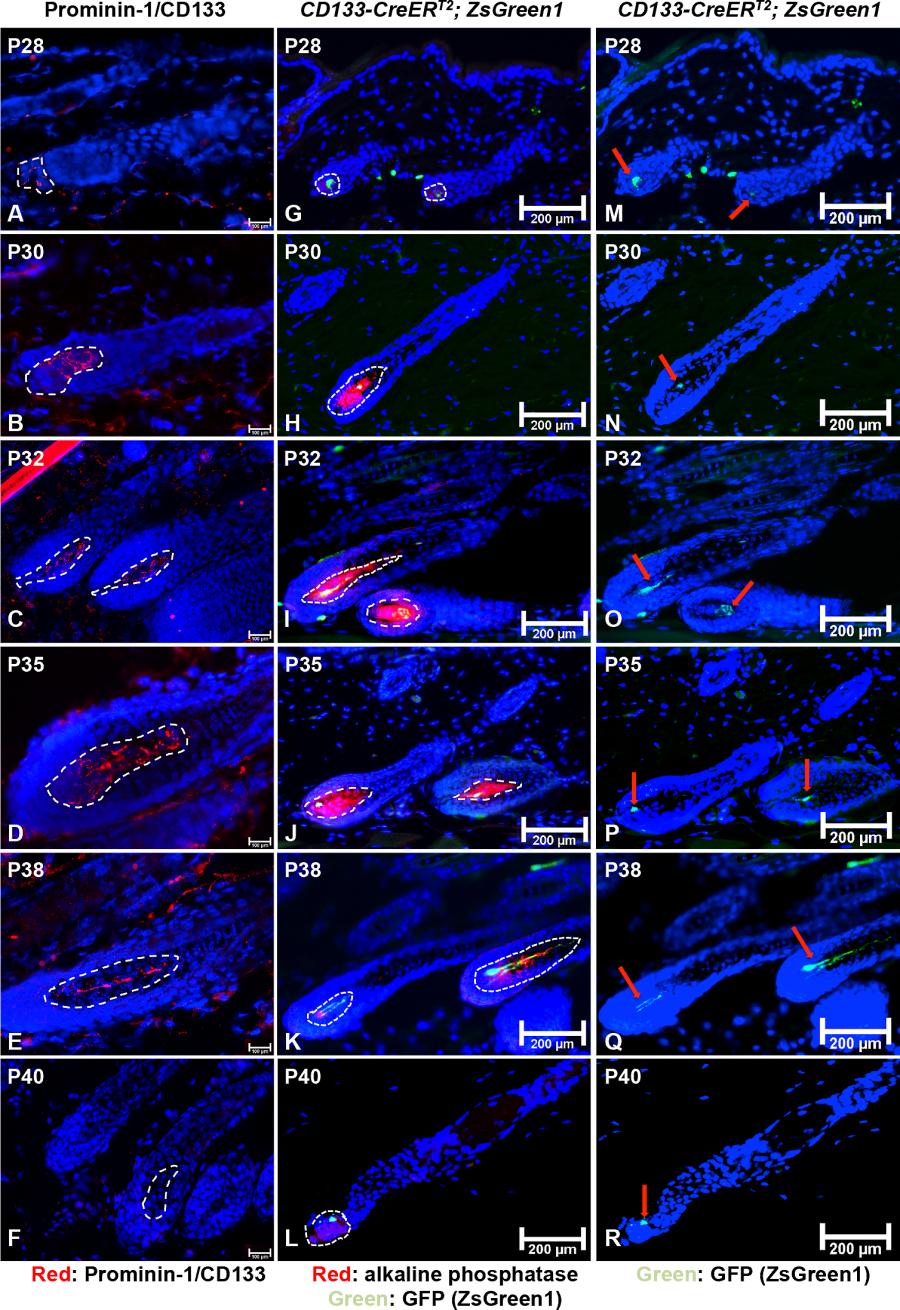
Endogenous CD133 expression in postnatal murine hair follicles and lineage tracing using *ZsGreen1* reporter mice. (**A-F**) Frozen sections of back skin biopsies of normal C57BL/6 mice at each indicated age were immunostained with an anti-CD133 antibody. CD133 was expressed in a subpopulation of cells in the DP (circled by white dashed line) of P28 to P38 anagen hair follicles (**A-E**) but not in hair follicles that in catagen or later (**F**). (**G-L**) Lineage tracing of CD133+ DP cells using *CD133-CreER*^*T2*^; *ZsGreen1* reporter mice. The DP is co-stained for the expression of alkaline phosphatase (red), which is a specific DP marker. GFP+ cells were first detected in DPs at P28 (**G**) and present throughout the entire anagen (**H-K**). An average of 20% of telogen hair follicles contained a small population of GFP+ cells in the DP (**L**). (**M**-**R**) Images of ZsGreen1 expression (green) in CD133+ DP cells during the hair cycle without staining for alkaline phosphatase expression. Green fluorescent DP cells were indicated with red arrows. For every indicated mouse age, at least three mice were analyzed. Scale bar: (A-F), 100 μm; (G-R), 200 μm

To examine the expression of endogenous CD133 in the DP during postnatal hair cycle, we analyzed mouse back skin collected from different postnatal stages by immunofluorescent staining. As shown in Fig 2A, only weak expression of CD133 could be seen in the DP at P28 when hair follicle enters early anagen (circled by white dashed line). Anagen stage of P28 hair follicles was confirmed by immunostaining for Ki67 expression (S2A Fig, green color). At P30, CD133 expression was clearly detected in a subpopulation of DP cells (Fig 2B). Expression of CD133 continued to be present in the DP (Fig 2C–2E) until P40 when CD133 expression was lost in the DP (Fig 2F). Weak CD133 expression was seen in the dermis for all stages of skin samples (red spots outside hair follicles) while no expression of CD133 was observed in the interfollicular epidermis. By contrast with a previous report [8], we did not detect CD133 expression in the hair follicle bulge at each time point. Interestingly, expression of β-catenin and CD133 coincide in the DP, suggesting that Wnt/β-catenin signaling could be important for the function of CD133+ DP cells.

### In vivo tracing of CD133-positive dermal papilla cells during the hair growth cycle

To genetically target CD133+ DP cells, we used a *Prom1-CreER*^*T2*^*-IRES-nLacZ (CD133-CreER*^*T2*^*)* mouse line, in which the expression of a fusion protein (CreER) combining the Cre recombinase and a mutated ligand-binding domain of the human estrogen receptor is placed under the control of the CD133 promoter [10]. Therefore, Cre recombinase encoded by the *CreER* transgene is specifically expressed in CD133+ DP cells in mouse skin, and can only be activated to induce site-specific gene recombination when the mice are treated with tamoxifen or 4-hydroxytamoxifen (4-OHT) [21].

To perform genetic lineage tracing, we crossed *CD133-CreER*^*T2*^ mice with *Rosa-CAG-LSL-ZsGreen1* reporter mice (*ZsGreen1*, Jax 007906) [22]. *ZsGreen1* mice harbor a loxP-flanked STOP cassette, which prevents the transcription of a CAG promoter-driven green fluorescent protein (ZsGreen1) in the *Rosa26* locus unless Cre recombinase is activated to remove the STOP cassette. We will refer to double transgenic reporter mice as *CD133-CreER*^*T2*^; *ZsGreen1*.

*CD133-CreER*^*T2*^; *ZsGreen1* mice were treated with tamoxifen by intraperitoneal injection to induce activation of Cre recombinase from P19 to P25. Skin samples were taken from the mid-dorsal region, embedded in tissue embedding medium (O.C.T) and serial sectioned for detection of green fluorescent cells. Frozen sections were co-stained for the expression of alkaline phosphatase (AP), a specific DP marker [23]. At least three mice were analyzed at each time point. As shown in Fig 2G, weak green fluorescence could be detected in the DP at P28, which corresponds to the time point when we can identify weak CD133 expression. Anagen stage of P28 hair follicles was confirmed by immunostaining for Ki67 expression (S2B Fig, red). Following the growth of hair follicle, ZsGreen1+ DP cells (green) could be consistently detected as a small subpopulation in the DP (red) from anagen (P30, P32, P35, P38) to catagen (P40) (Fig 2H–2L). This observation is slightly different from the lack of CD133 expression in the DP of catagen hair follicles (Fig 2F) or later. The major reason accounting for this discrepancy is that the expression of ZsGreen1 is driven by the *Rosa26* promoter and not directly by the CD133 promoter. Therefore, after the STOP cassette is removed by Cre recombinase, progenies from CD133+ DP cells may no longer express CD133 but still generate ZsGreen1 protein, which then labels the CD133+ DP lineage. Green fluorescent ZsGreen1 expression was clearly seen in the DP without AP co-staining (Fig 2M–2R).

Overall, at each time point examined, at least 50% of hair follicle DPs contained a small population of ZsGreen1+ cells from P28 to P40. We did not detect any green fluorescent cells in back skin in the absence of tamoxifen treatment (data not shown). Consistent with other reports, we only detected a very limited number of ZsGreen1+ cells in the interfollicular dermis and dermal sheath, suggesting that the *CD133-CreER*^*T2*^ mouse line is a specific and suitable model for genetic manipulation of target gene expression in CD133+ DP cells and their descendants. Worth to note, no green cells were seen in the hair bulge area.

### Generation of a triple transgenic animal model that allows expression of a constitutively active form of β-catenin (ΔN-β-catenin) in CD133+ dermal papilla cells

**Fig 3:**
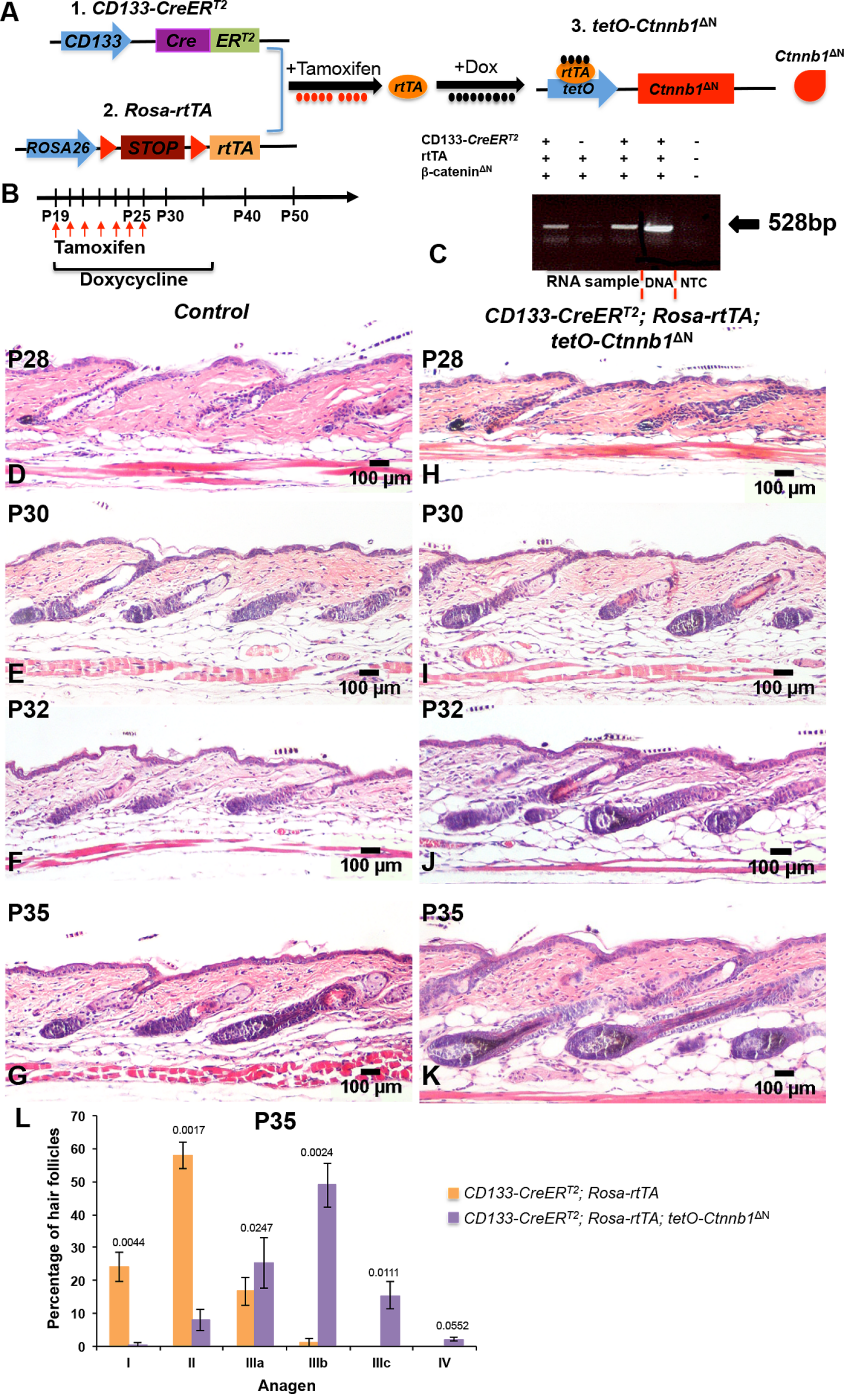
**Expression of ΔN-β-catenin in CD133+ DP cells accelerates spontaneous hair anagen** (**A**) Illustration of the triple transgenic mouse model. Expression of ΔN-β-catenin in CD133+ DP cells requires administration of both tamoxifen, which induces rtTA expression, and doxycycline, which forms a complex with rtTA to bind to the *tetO* promoter. (**B**) The time scheme for mouse induction. (**C**) Detection of ∆N-β-catenin expression in *CD133-CreER*^*T2*^; *Rosa-rtTA; tetO-Ctnnb1*^ΔN^ mutant mice by RT-PCR. Back skin biopsies from *CD133-CreER*^*T2*^; *Rosa-rtTA; tetO-Ctnnb1*^ΔN^ mutant mice. (**H-K**) and control littermates (**D-G**) were stained with H&E and photographed at indicated stages. Scale bars: 100 μm. (**L**) The numbers of hair follicles at P35 were counted and compared between *CD133-CreER*^*T2*^; *Rosa-rtTA; tetO-Ctnnb1*^ΔN^ mutant mice and control littermates (mean ± s.d.). A minimum of three skin biopsies from three pairs of mutant and control mice was analyzed. Two-tailed paired Students t-test was employed to calculate statistical significance.

We generated a triple transgenic mouse line, *CD133-CreER*^*T2*^; *Rosa-rtTA; tetO-Ctnnb1*^ΔN^, which allows for constitutive expression of ΔN-β-catenin in CD133+ DP cells. In contrast to previous studies, ΔN-β-catenin expression is controlled by a doxycycline-inducible *tetO* promoter, rather than the endogenous β-catenin promoter, in our model. To activate this promoter, reverse tet transactivator (rtTA) has to be expressed and bind doxycycline. However, in the *Rosa-rtTA* transgene, a loxP-flanked STOP cassette prevents the transcription of a CAG promoter-driven rtTA from the Rosa26 locus [24]. As shown in Fig 3A, expression of *rtTA* is initiated when a Cre recombinase is introduced to remove the STOP cassette. The activation of Cre recombinase encoded by the *CreER* transgene in *CD133-CreER*^*T2*^ *mice* requires administration of tamoxifen or 4-hydroxytamoxifen (4-OHT) [10, 21].

### Expression of ΔN-β-catenin in CD133+ DP cells accelerates anagen phase

As shown in Fig 3B, we induced expression of rtTA in *CD133-CreER*^*T2*^; *Rosa-rtTA; tetO-Ctnnb1*^ΔN^ mice by intraperitoneal injection of tamoxifen starting at P19 when hair follicles enter the first postnatal hair telogen phase, which normally is very short (lasts one to two days), and continuing for 7 days until P25. Experimental mice were maintained on doxycycline chow at the same time and beyond, resulting in the expression of ΔN-β-catenin in CD133+ cells. Control littermates were either of genotypes *Rosa-rtTA; tetO-Ctnnb1*^ΔN^ or *CD133-CreER*^*T2*^*; Rosa-rtTA*. RT-PCR assays showed that only triple transgenic mice expressed ΔN-β-catenin protein upon the simultaneous administration of tamoxifen and doxycycline (Fig 3C). Dorsal skin was harvested at P28, P30, P32, P35, P40, P42, P45 and P50 for detailed analyses of the natural hair growth cycle. Consistent with published reports [17], treatment with tamoxifen and doxycycline alone led to an anagen onset delay of 2 to 3 days. Therefore, timing of the hair cycle stages in our study differ slightly from those in untreated mice [1].

At P28 when hair follicles entered anagen, no obvious difference in hair follicle morphology could be seen between *CD133-CreER*^*T2*^*; Rosa-rtTA; tetO-Ctnnb1*^ΔN^ triple transgenic mice (Fig 3H) and control littermates (Fig 3D). At P30, hair follicles in triple transgenic mice appeared slightly more advanced (Fig 3I) than those in controls (Fig 3E). Starting from P32, hair follicles in triple transgenic mice exhibited a quicker hair cycle progression (Fig 3J–3K) than those in controls (Fig 3F–3G), suggesting that constitutive expression of ΔN-β-catenin in CD133+ DP cells induced a significant acceleration of hair growth. This is in line with the fact that endogenous CD133 expression and subsequently expression of Cre activity in our model system is absent at anagen onset but initiated shortly after. As shown in Fig 3L, at P35, an average of 57% ± 3.96% hair follicles were in anagen II stage in control mice while a mean of 48% ± 6.77% had already entered the anagen IIIb stage in *CD133-CreER*^*T2*^; *Rosa-rtTA; tetO-Ctnnb1*^ΔN^ mice.

To further confirm the expression of ΔN-β-catenin in CD133+ DP cells accelerates hair growth anagen, we treated *CD133-CreER*^*T2*^; *Rosa-rtTA; tetO-Ctnnb1*^ΔN^ mutant mice and their respective littermate controls from P50 (telogen) with tamoxifen while on Dox diet, and plucked hair at P52 to induce a synchronized growth phase. As shown in S3 Fig, at 5 days postplucking (PP5d), there was no clear difference between hair follicles in *CD133-CreER*^*T2*^; *Rosa-rtTA; tetO-Ctnnb1*^ΔN^ mutant mice (S3F Fig) and controls (S3A Fig). This observation is in agreement with the report that CD133 expression would not happen until 6 days after depilation [7]. 8 and 10 days after plucking (PP8d and PP10d), when both hair follicles reached anagen, hair follicles that expressed ΔN-β-catenin (S3G and S3H Fig) appeared at a much-advanced stage than control hair follicles (S3B and S3C Fig). 15 days after depilation (PP15d), hair follicles in both mutants and controls entered catagen phase, however, control hair follicles (S3D Fig) regressed much faster than hair follicles in *CD133-CreER*^*T2*^; *Rosa-rtTA; tetO-Ctnnb1*^ΔN^ mutant mice (S3I Fig). Consistent with our observation in the natural hair cycle, when both hair follicles entered telogen stage at P50, 20 days after depilation and induced hair cycling, hair follicles in *CD133-CreER*^*T2*^*; Rosa-rtTA; tetO-Ctnnb1*^ΔN^ triple transgenic mice (S3J Fig) and control littermates (S3E Fig) advanced to telogen.

**Fig 4:**
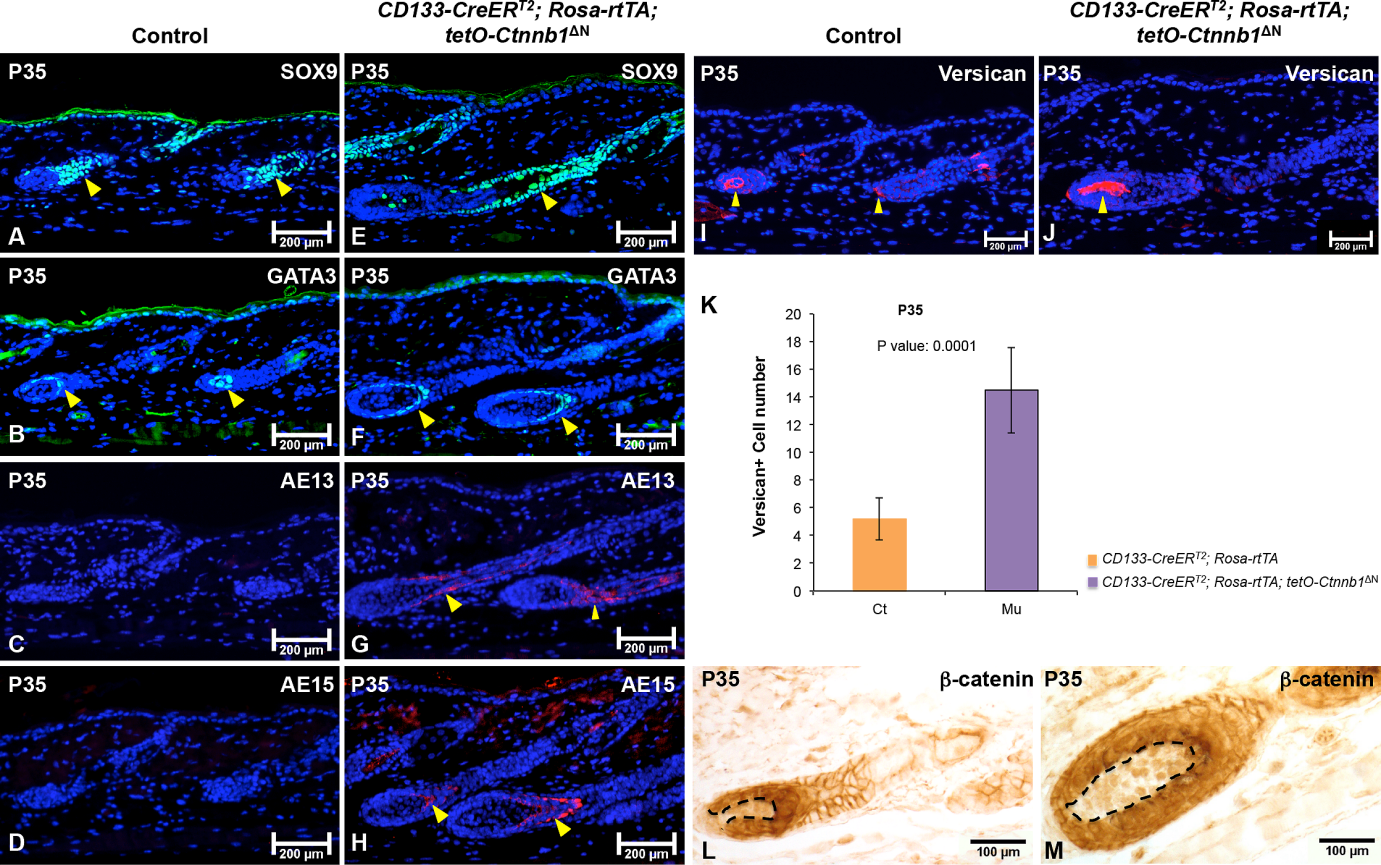
Expression of ΔN-β-catenin in CD133+ DP cells accelerates hair follicle differentiation. 5-μm-thick paraffin sections from P35 *CD133-CreER*^*T2*^; *Rosa-rtTA; tetO-Ctnnb1*^ΔN^ mutant mice and control littermates were processed for immunofluorescence staining of following markers: Sox9 for outer root sheath (control: **A**; mutant: **E**); Gata3 for inner root sheath (control: **B**; mutant: **F**); AE13 and AE15 for hair keratins (control: **C**, **D**; mutant: **G**, **H**); versican for anagen DP (control: **I**; mutant: **J**). Sections were nuclear counterstained with DAPI (blue). **K**. The numbers of versican+ DP cells in each hair follicle were counted and compared between *CD133-CreER*^*T2*^; *Rosa-rtTA; tetO-Ctnnb1*^ΔN^ mutant mice and control littermates (mean ± s.d). A minimum of three skin biopsies from three pairs of mutant and control mice was analyzed. Two-tailed paired Student’s t-test was employed to calculate statistical significance. (**L-M**) β-catenin expression in hair follicle was examined by immunohistochemistry. Images shown are representative of at least three replicates at each indicated age. Scale bars: 200 μm for A-J; 100 μm for L-M.

To determine whether the expression of various hair follicle markers, including Sox9 (outer root sheath), Gata3 (inner root sheath) [25], AE13 (hair shaft cortex keratin) [26], and AE15 (medulla) [26], were consistent with the observed phenotypes in *CD133-CreER*^*T2*^; *Rosa-rtTA; tetO-Ctnnb1*^ΔN^ mice, we analyzed their expression patterns by immunostaining. At P35, expression of Sox9 and Gata3 in the inner and outer root sheaths, respectively, was higher in mutant hair follicles (Fig 4E and 4F) than in control hair follicles (Fig 4A and 4B). Immunostaining for AE13 and AE15, markers for the hair shaft, was present in hair follicles in *CD133-CreER*^*T2*^; *Rosa-rtTA; tetO-Ctnnb1*^ΔN^ mice (Fig 4G and 4H), but, remarkably, their expression was missing in control hair follicles (Fig 4C and 4D). We also examined the expression of versican, a marker for DP cells of anagen hair follicles [27], by immunostaining. As shown in Fig 4J, the size of the versican+ DP cell population was much increased in *Ctnnb1*^ΔN^ expressing DPs compared with control DPs (Fig 4I). As shown in Fig 4K, at P35, an average of 5 ± 2 versican+ DP cells was found in each control hair follicle while a mean of 15 ± 3 versican+ DP cells existed in *CD133-CreER*^*T2*^; *Rosa-rtTA; tetO-Ctnnb1*^ΔN^ hair follicle. β-catenin immunohistochemistry clearly demonstrated up-regulated expression of β-catenin protein in mutant DPs (Fig 4M).

### Expression of ΔN-β-catenin in CD133+ DP cells does not prevent regression of hair follicles

**Fig 5:**
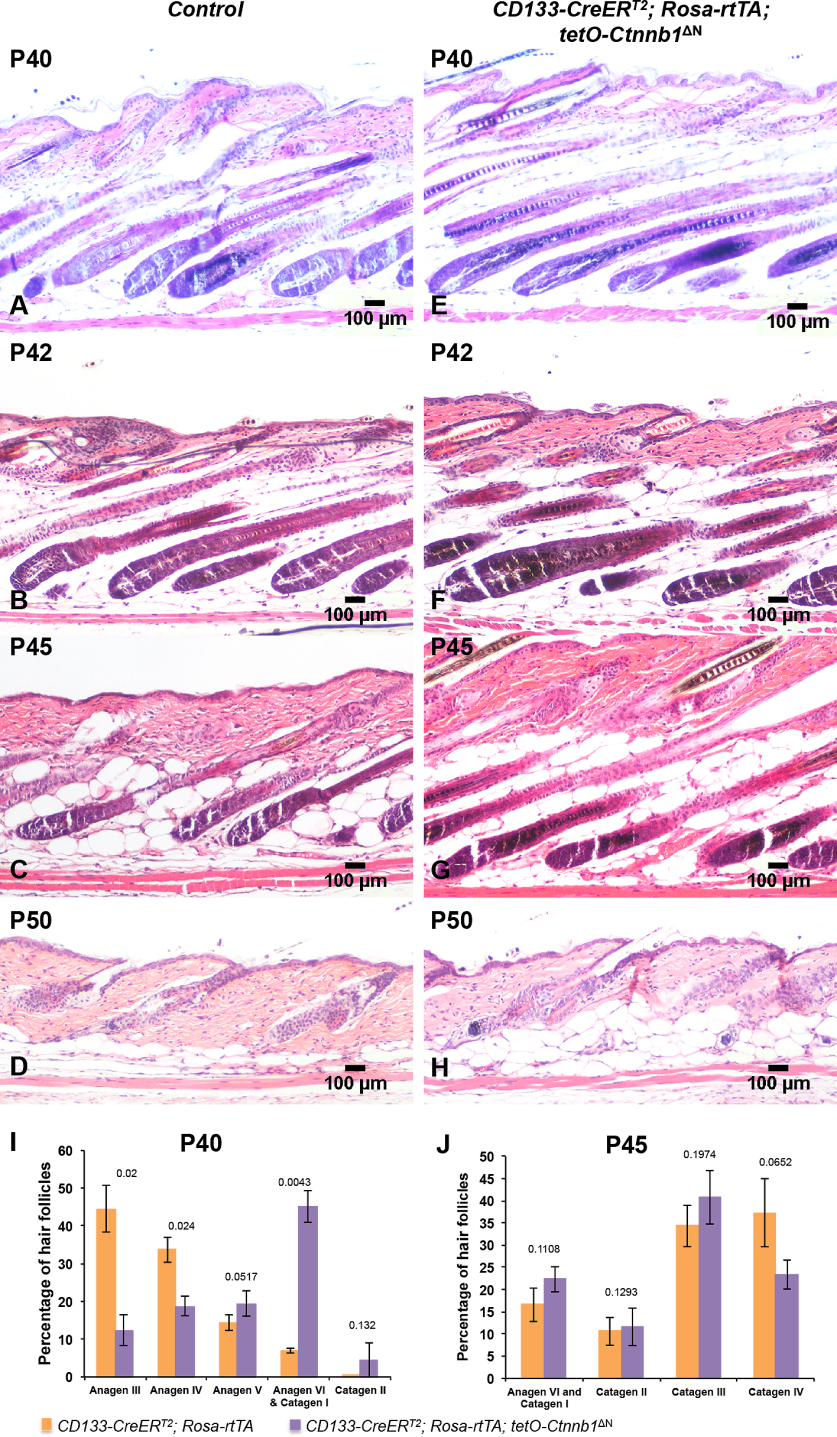
Expression of ΔN-β-catenin in CD133+ DP cells does not block hair follicle regression. Back skin biopsies from *CD133-CreER*^*T2*^; *Rosa-rtTA; tetO-Ctnnb1*^ΔN^ mutant mice (**H-K**) and control littermates (**D-G**) were stained with H&E and photographed at indicated stages. Scale bars: 100 μm. (**L**) The numbers of hair follicles at P35 were counted and compared between *CD133-CreER*^*T2*^; *Rosa-rtTA; tetO-Ctnnb1*^ΔN^ mutant mice and control littermates (mean ± s.d.). A minimum of three skin biopsies from three pairs of mutant and control mice was analyzed. Two-tailed paired Student’s t-test was employed to calculate statistical significance.

Control (Fig 5A) and *CD133-CreER*^*T2*^; *Rosa-rtTA; tetO-Ctnnb1*^ΔN^ (Fig 5B) hair follicles appeared to be in comparable hair cycle stages at P40 although mutant hair follicles were still slightly more advanced. By counting, a mean of 78% ± 5.79% hair follicles reached anagen III and IV stages in control mice while over 45% ± 4.19% of hair follicle progressed into anagen VI and catagen stages in *CD133-CreER*^*T2*^; *Rosa-rtTA; tetO-Ctnnb1*^ΔN^ mice (Fig 5I), indicating hair follicles reached full anagen stage in control littermates while hair follicles in *CD133-CreER*^*T2*^; *Rosa-rtTA; tetO-Ctnnb1*^ΔN^ mice gradually entered the later anagen stage and catagen.

However, starting P42, control hair follicles (Fig 5B) regressed quicker than hair follicles in *CD133-CreER*^*T2*^; *Rosa-rtTA; tetO-Ctnnb1*^ΔN^ mice did (Fig 5F). At P45, there were more catagen hair follicles in control mice (Fig 5C) than in *CD133-CreER*^*T2*^; *Rosa-rtTA; tetO-Ctnnb1*^ΔN^ mice (Fig 5G). Counting numbers of hair follicles that were present at different hair cycle stages between control and *CD133-CreER*^*T2*^; *Rosa-rtTA; tetO-Ctnnb1*^ΔN^ supported this trend (Fig 5J). At P50, most of hair follicles in both *CD133-CreER*^*T2*^; *Rosa-rtTA; tetO-Ctnnb1*^ΔN^ mice and control littermates were in telogen, suggesting CD133+ cells only function in the early-to-mid anagen stages and are unable to prevent the entry of hair follicles into catagen and telogen even in the presence of activated β-catenin. β-catenin immunostaining confirmed that there was no overexpression of nuclear β-catenin in mutant DP (S4B Fig).

### Rapid progression of mutant hair follicle cycling in *CD133-CreER*^*T2*^*; Rosa-rtTA; tetO-Ctnnb1*^*ΔN*^ mice is associated with greater matrix cell proliferation

**Fig 6:**
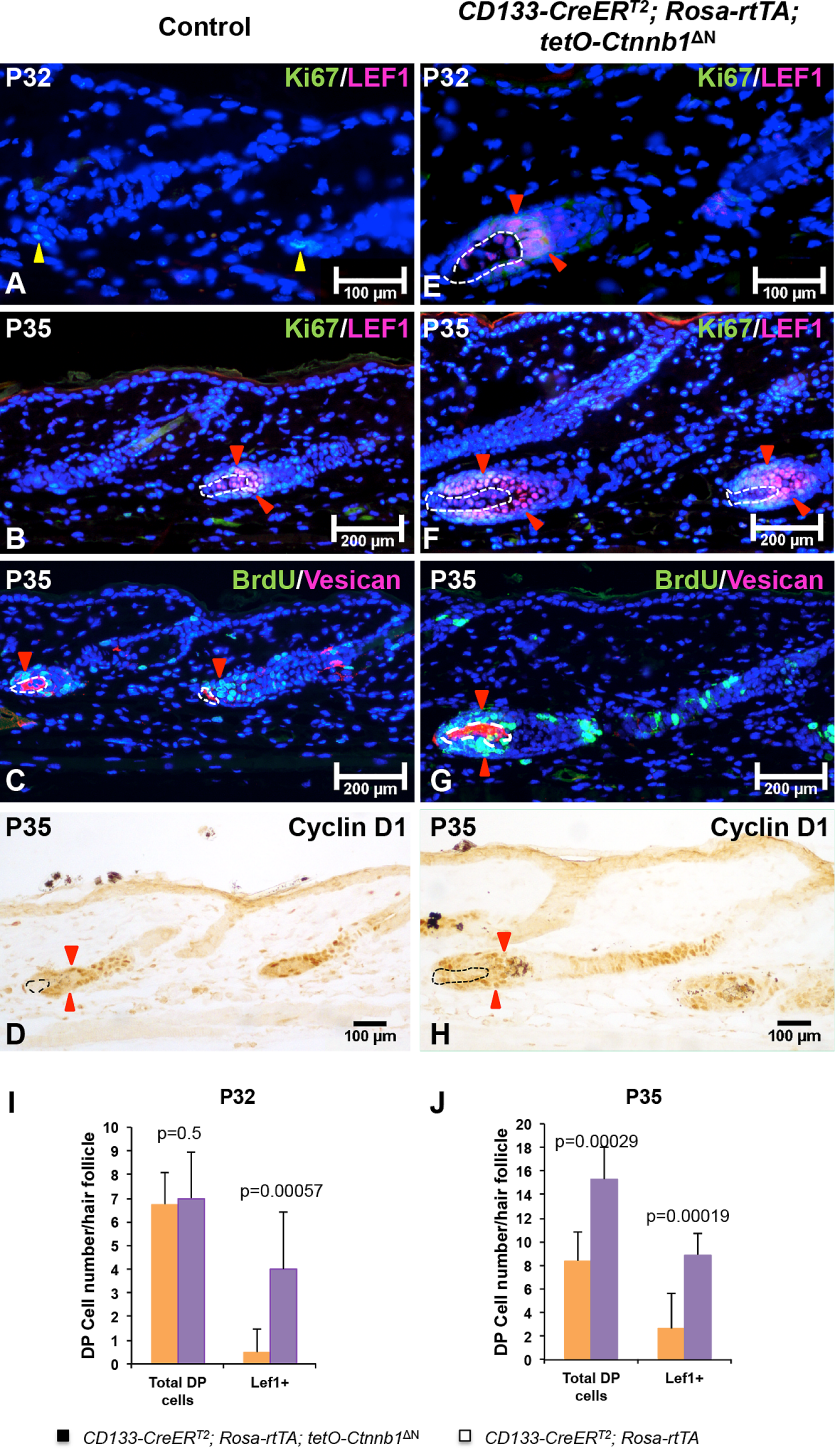
Increased proliferation in both matrix keratinocytes and DP cells in *CD133-CreER*^*T2*^; *Rosa-rtTA; tetO-Ctnnb1*^ΔN^ hair follicles. Co-expression of Ki67 (green) and Lef1 (red) was examined in skin samples collected from *CD133-CreER*^*T2*^; *Rosa-rtTA; tetO-Ctnnb1*^ΔN^ mice and control littermates at P32 (control: **A**; mutant: **E**) and P35 (control: **B**; mutant: **F**). Paraffin slides from BrdU-incorporated skin biopsies co-stained with anti-versican antibody showing enlarged DP compartment in hair follicles of from *CD133-CreER*^*T2*^; *Rosa-rtTA; tetO-Ctnnb1*^ΔN^ mice with increased numbers of proliferating DP cells and matrix cells (**G**) than in littermate controls (**C**). Cyclin D1 staining indicated accelerated cell cycle progression in hair matrix cells when ΔN-β-catenin was expressed in CD133+ DP cells (**H**). Images shown are representative of at least three replicates at each indicated age. Total cell number (**I**) and number of Lef1+ DP cells (**J**) in each hair follicle at P28 and P35 were counted and compared between *CD133-CreER*^*T2*^; *Rosa-rtTA; tetO-Ctnnb1*^ΔN^ mice and control littermates. 20 hair follicles were counted for each mouse (mean ± s.d.). For each indicated age, at least three pairs of mutant and control mice were used. Scale bars: 200 μm for A-C and E-G; 100 μm for D and H.

To determine the underlying causes of accelerated hair growth in *CD133-CreER*^*T2*^; *Rosa-rtTA; tetO-Ctnnb1*^ΔN^ mice, we evaluated the expression of specific markers for different cell populations in hair follicles, including Ki67 for hair matrix cell proliferation and Lef1 for hair shaft progenitor cells [28]. Immunofluorescent co-staining for Ki67 and Lef1 did not show any difference between mutant and control littermates (data not shown). At P32, there was intense labeling of Ki67+ matrix cells and Lef1+ hair shaft precursor cells in mutant hair follicles (Fig 6E). Ki67/Lef1 double-positive cells were present in hair follicles of *CD133-CreER*^*T2*^; *Rosa-rtTA; tetO-Ctnnb1*^ΔN^ mice (indicated by red triangles in Fig 6E), which were clearly in advanced anagen stages. By contrast, there was a lack of Lef1+ hair shaft precursor cells in control hair follicles at this stage (Fig 6A). At P35, Lef1+ cells appeared in control hair follicles (Fig 6B), while there were greatly increased numbers of Ki67+ matrix cells and Lef1+ hair shaft precursor cells in mutant hair follicles (Fig 6F). Immunostaining for incorporated BrdU also showed increased proliferation in the hair matrix of mutant hair follicles at P35 (Fig 6G) as compared with control follicles (Fig 6C). Cyclin D1 is a direct target of Wnt/β-catenin signaling and promotes cell cycle progression from late G1 to S phase [29]. Mutant hair follicles showed increased expression of the proliferation marker cyclin D1 in matrix cells surrounding the DP (indicated by red triangles in Fig 6H), suggesting proliferation of matrix cells in *CD133-CreER*^*T2*^; *Rosa-rtTA; tetO-Ctnnb1*^ΔN^ hair follicles was significantly increased.

### Expression of ΔN-β-catenin in CD133+ DP cells leads to increased DP cell proliferation

We next determined the impact of ΔN-β-catenin on the DP compartment. As shown in Fig 6E, nuclear Lef1 expression could be clearly identified in the DP of *CD133-CreER*^*T2*^; *Rosa-rtTA; tetO-Ctnnb1*^ΔN^ hair follicles at P32 (circled by white dashed line in Fig 6E). At P35, there were more Lef1+ cells in mutant DPs (circled by white dash line in Fig 6F) than control DPs (Fig 6B). The numbers of Lef1+ cells were counted and compared between *CD133-CreER*^*T2*^; *Rosa-rtTA; tetO-Ctnnb1*^ΔN^ mutant and control hair follicles. As shown in Fig 6I, the number of Lef1+ DP cells in each hair follicle was greatly increased in mutant mice while the total number of DP cells remained unchanged at P32. At P35, the numbers of both total and Lef1+ DP cells were higher in mutant hair follicles than in control hair follicles (Fig 6J). These data together confirmed that expression of ΔN-β-catenin in CD133+ DP cells led to up-regulated Wnt/β-catenin signaling in DP cells.

The DP comprises a relatively static population of cells, which normally do not show much proliferative activity during the hair growth cycle. Surprisingly, we could clearly identify BrdU-positive DP cells, which co-stained with versican, in *CD133-CreER*^*T2*^; *Rosa-rtTA; tetO-Ctnnb1*^ΔN^ hair follicles (Fig 6G). There were no BrdU and versican double-positive DP cells in control hair follicles (Fig 6C), suggesting that upregulated β-catenin activity promoted DP cell proliferation. Cyclin D1-positive DP cells could also be seen in the mutant DP (indicated by green arrows in Fig 6H). In line with this finding, the number of DP cells was increased in mutant hair follicles (Fig 6J). Analysis of skin histology showed that the mean size of DPs in mutant *CD133-CreER*^*T2*^; *Rosa-rtTA; tetO-Ctnnb1*^ΔN^ hair follicles (Fig 3H–3K) was increased compared with controls during early anagen stages (Fig 3D–3G).

## Discussion

Here we report a novel hair growth-promoting role for Wnt/β-catenin signaling in CD133+ DP cells. Although the DP is considered to be static and normally shows little proliferation, the composition of the DP is heterogeneous and dynamic. In murine DPs, distinct DP cell subpopulations have been identified based on the expression of unique markers, including CD133 and Sox2 [14, 30]. CD133+ DP cells possess special abilities to induce hair follicle regeneration [7]. Our analysis of CD133 expression in the DP is consistent with prior reports that CD133+ DP cells can only be transiently identified in the DP during the early anagen stages but is not present during the telogen-to-anagen transition or at anagen onset [7, 19]. This observation suggests that CD133+ DP cells contribute to hair growth after anagen onset, but may not play a role in anagen induction.

To genetically trace CD133+ DP cells *in vivo*, we used a *CD133-CreER*^*T2*^*; ZsGreen1* reporter mouse. No green fluorescence was detected in the DP at the onset of the anagen phase of the hair growth cycle from P21 to P25. Starting from P28, a small subset of green fluorescent cells could be clearly detected in the DP. This observation is consistent with CD133 expression pattern determined by immunostaining, indicating that CD133+ cells transiently appear during the early stages of anagen but are not present at anagen onset. No green cells were seen surrounding the DP or in the DS. However, we observed a very small population of green cells in about 10–15% of telogen DPs. This observation indicates that some CD133+ lineage DP cells survive catagen and telogen. We, therefore, speculate that CD133+ DP cells are generated primarily *in situ* in the DP after the initiation of anagen.

Importantly, we did not find any green cells in the hair follicle bulge as reported previously. This deficiency suggests a lack of CD133-driven Cre activity in the bulge stem cells, which is further supported by our observation that the hair growth phenotype induced by ΔN-β-catenin expression in CD133+ DP cells only appeared after anagen onset. This phenotype was strongly supported by a recent publication from the Watt group, in which they did not report any early phenotype when overexpressing a stabilized form of β-catenin in CD133+ DP cells [19]. Furthermore, if the CD133 promoter were active in the bulge, considering the strong effects of Wnt/β-catenin signaling, we would understandably expect to see some phenotypes at the onset of hair cycling. Previously, we showed strong and dramatic hair phenotypes when β-catenin was overexpressed in epidermal keratinocytes, including the bulge stem cells [31]. Taken together, our data demonstrate that the *CD133-CreER*^*T2*^ transgene is specifically activated during the anagen phase in a subset of DP cells and shows very little if any additional activity in other skin cell populations.

To bypass the expression limitation of using the endogenous β-catenin promoter, we have designed a triple transgenic mouse model that expresses a ΔN-β-catenin protein under the control of a *tetO* promoter. In this model, ΔN-β-catenin is transcribed when rtTA is expressed in CD133+ DP cells and their descendants, which occurs when doxycycline is provided. Our decision to treat experimental mice between P19 and P25 when hair follicles entered anagen is based on a combination of technical considerations, including CD133 expression, maximal recombination efficiency, the length of time needed for TAM-induced Cre-loxP recombination to occur, and side effects of TAM treatment. Particularly, for our triple transgenic system, once Cre recombinase is activated by tamoxifen and generates rtTA, rtTA still needs to bind to doxycycline in order to induce ΔN-β-catenin expression. This complex *in vivo* metabolic process requires time to complete. Therefore, administrating tamoxifen when CD133 expression is at the peak may not allow the system to function at the required time points and to its maximum. Most importantly, it has been shown by several groups that tamoxifen or the tamoxifen metabolite 4-OHT function for a prolonged period after treatment [24, 32]. Furthermore, as we described above, tamoxifen has been shown to delay the hair growth cycle onset. Administrating tamoxifen earlier between P19 and P25 not only gives the system enough time to metabolized tamoxifen and function, and also minimizes adverse effects caused by tamoxifen.

Our model system produces a clear hair growth phenotype of accelerated anagen. Nevertheless, we did not observe accelerated anagen onset, which is consistent with the notion that CD133+ DP cells are not present at anagen onset and do not play a role in the transition from telogen to anagen. However, further analysis showed that there was increased proliferation in matrix keratinocytes following the expression of ΔN-β-catenin in CD133+ DP cells. This observation suggests that signaling molecules secreted by CD133+ DP cells in response to β-catenin activation function in a paracrine manner to stimulate hair matrix cells to proliferate and differentiate.

In the adult murine hair cycle, hair follicles enter catagen and telogen when matrix keratinocytes undergo apoptosis, and the entire non-permanent part of the hair follicle degrades. Interestingly, we did not observe any obvious differences in the timing of catagen and telogen between normal hair follicles and hair follicles that express ΔN-β-catenin in CD133+ DP cells. This is consistent with the loss of CD133 expression in DP cells when hair follicles enter the regression stage. However, as discussed earlier, some progeny cells of the CD133+ lineage were detected in catagen hair follicles (no longer expressing CD133), suggesting expressing ΔN-β-catenin alone is not enough to sustain the ability of these cells to prevent the anagen to catagen transition. A potential explanation for failing to prevent catagen entry is the amount of ΔN-β-catenin produced by limited number of CD133+ DP progenies is too low to make any realistic impact although highly unlikely. At P50, we couldn’t detect any overexpression of nuclear β-catenin in the DP of mutant hair follicles, suggesting those CD133 progeny cells are eventually lost in the DP. Another possible reason is the lack of hair shaft precursor cells at the end of normal anagen, which are normally provided by transit-amplifying (TA) cells derived from HFSCs. Controlled generation of TA cells in the epithelial compartment may prove to be a rate-limiting factor that cannot be overcome by expression of ΔN-β-catenin in CD133+ DP cells.

Since only a small percentage of cells in the DP is CD133+, what is the role and function of CD133- DP cells in the hair follicle regenerative cycle? We speculate that CD133+ DP cells are only needed after anagen onset and function to stimulate CD133- DP cells for the regeneration of hair follicle structure by paracrine signaling downstream of β-catenin activation. As soon as the hair-building process begins, CD133+ DP cells may no longer be needed. This notion is supported by our observation that both total cell number and the number of Lef1+ cells in the DP were increased when ΔN-β-catenin was expressed in CD133+ DP cells. However, there was no significant increase in the number of CD133+ cells, suggesting that expression of ΔN-β-catenin in CD133+ DP cells triggered proliferation of CD133- DP cells via intercellular signaling interactions. By BrdU labeling, we could see strong BrdU staining in the DP of *CD133-CreER*^*T2*^; *Rosa-rtTA; tetO-Ctnnb1*^ΔN^ hair follicles, indicating that proliferation of DP cells was increased. However, we cannot exclude the possibility that increased DP size was caused by accelerated proliferation of matrix keratinocyte and hair follicle cycling. Identifying the underlying mechanisms will be an interesting area for further study in future.

Taken together, our results reveal that forced β-catenin activation enhances the hair-promoting ability of CD133+ DP cells. The downstream secreted factors that facilitate interactions among CD133+ DP cells, CD133- DP cells and matrix keratinocytes to promote hair growth have not been fully characterized and will be an important subject for future exploration.

## Supporting Information

S1 Fig Nuclear β-catenin is detected in the DP during anagen. Nuclear β-catenin is detected in the DP during anagen. (**A-F**) Skin biopsies of normal C57BL/6 mice were collected at the indicated age and processed for paraffin sections. Expression of β-catenin (brown color) was visualized by immunohistochemistry. The DP was circled by white dashed lines in each hair follicle. Scale bar: 100 μm.
(TIF)

S2 Fig Keratinocytes in P28 hair follicles are Ki67 positive. P28 skin biopsies of normal C57BL/6 mice (A) and *CD133-CreER*^*T2*^; *ZsGreen1* mice (**B**) were analyzed for Ki67 expression by immunofluorescence staining. The DP was circled by white dashed lines in each hair follicle. Scale bar: 100 μm.
(TIF)

S3 Fig Expression of ΔN-β-catenin in CD133+ DP cells accelerates depilation-induced hair growth anagen. *CD133-CreER*^*T2*^; *Rosa-rtTA; tetO-Ctnnb1*^ΔN^ mutant mice and control littermates were treated with tamoxifen from P50 for 7 days while on Dox diet, and their mid-dorsal hairs were plucked at each indicated age. Back skin biopsies from depilated areas of *CD133-CreER*^*T2*^; *Rosa-rtTA; tetO-Ctnnb1*^ΔN^ mutant mice (**F-J**) and control littermates (**A-E**) were process for paraffin sections and stained with H&E. A minimum of three skin biopsies from three pairs of mutant and control mice was analyzed. Two-tailed paired Student’s t-test was employed to calculate statistical significance. Scale bars: 100 μm.
(TIF)

S4 Fig Nuclear expression of β-catenin is not detected in the DP of *CD133-CreER*^*T2*^; *Rosa-rtTA; tetO-Ctnnb1*^ΔN^ mice during telogen. 5-μm-thick paraffin sections from P50 *CD133-CreER*^*T2*^; *Rosa-rtTA; tetO-Ctnnb1*^ΔN^ mutant mice (**B**) and control littermates (**A**) were processed for β-catenin immunofluorescence staining. The DP was circled by white dashed lines in each hair follicle. Scale bar: 100 μm.
(TIF)

## References

[1] MuÈller-RoÈver S, Handjiski B, van der Veen C, Eichmhmdren’s Research Hospital f accelerated proliferation of matrix keratinocyte and hair follicle cycling.t CD133 expression, maximal recombination efficiency, the length of time needed for TAM-induc.

[2] Cotsarelis G, Sun TT, Lavker RM. Label-retaining cells reside in the bulge area of pilosebaceous unit: implications for follicular stem cells, hair cycle, and skin carcinogenesis. Cell. 1990;61(7):1329–37. Epub 1990/06/29. doi: 0092-8674(90)90696-C [pii]. . 2364430 10.1016/0092-8674(90)90696-c

[3] Greco V, Chen T, Rendl M, Schober M, Pasolli HA, Stokes N, et al A two-step mechanism for stem cell activation during hair regeneration. Cell Stem Cell. 2009;4(2):155–69. 10.1016/j.stem.2008.12.009 19200804 PMC2668200

[4] Nishimura EK. Melanocyte stem cells: a melanocyte reservoir in hair follicles for hair and skin pigmentation. Pigment Cell Melanoma Res. 2011;24(3):401–10. 10.1111/j.1755-148X.2011.00855.x . 21466661

[5] Yang CC, Cotsarelis G. Review of hair follicle dermal cells. J Dermatol Sci. 2010;57(1):2–11. Epub 2009/12/22. 10.1016/j.jdermsci.2009.11.005 20022473 PMC2818774

[6] Chi W, Wu E, Morgan BA. Dermal papilla cell number specifies hair size, shape and cycling and its reduction causes follicular decline. Development. 2013;140(8):1676–83. 10.1242/dev.090662 23487317 PMC3621486

[7] Ito Y, Hamazaki TS, Ohnuma K, Tamaki K, Asashima M, Okochi H. Isolation of Murine Hair-Inducing Cells Using the Cell Surface Marker Prominin-1//CD133. J Invest Dermatol. 2006;127(5):1052–60. doi: http://www.nature.com/jid/journal/v127/n5/suppinfo/5700665s1.html. 17185982 10.1038/sj.jid.5700665

[8] Charruyer A, Strachan LR, Yue LL, Toth AS, Cecchini G, Mancianti ML, et al CD133 Is a Marker for Long-Term Repopulating Murine Epidermal Stem Cells. Journal of Investigative Dermatology. 2012;132(11):2522–33. 10.1038/jid.2012.196 . 22763787 PMC3997791

[9] Gay DL, Yang CC, Plikus MV, Ito M, Rivera C, Treffeisen E, et al CD133 expression correlates with membrane beta-catenin and E-cadherin loss from human hair follicle placodes during morphogenesis. J Invest Dermatol. 2015;135(1):45–55. 10.1038/jid.2014.292 25010141 PMC4465595

[10] Zhu LQ, Gibson P, Currle DS, Tong Y, Richardson RJ, Bayazitov IT, et al Prominin 1 marks intestinal stem cells that are susceptible to neoplastic transformation. Nature. 2009;457(7229):603–U114. 10.1038/nature07589 . 19092805 PMC2633030

[11] Suetsugu A, Nagaki M, Aoki H, Motohashi T, Kunisada T, Moriwaki H. Characterization of CD133+ hepatocellular carcinoma cells as cancer stem/progenitor cells. Biochem Biophys Res Commun. 2006;351(4):820–4. Epub 2006/11/14. 10.1016/j.bbrc.2006.10.128 . 17097610

[12] Choi SA, Wang KC, Phi JH, Lee JY, Park CK, Park SH, et al A distinct subpopulation within CD133 positive brain tumor cells shares characteristics with endothelial progenitor cells. Cancer Lett. 2012;324(2):221–30. 10.1016/j.canlet.2012.05.026 . 22652175

[13] Fusi A, Reichelt U, Busse A, Ochsenreither S, Rietz A, Maisel M, et al Expression of the stem cell markers nestin and CD133 on circulating melanoma cells. J Invest Dermatol. 2011;131(2):487–94. 10.1038/jid.2010.285 . 20882037

[14] Driskell RR, Juneja VR, Connelly JT, Kretzschmar K, Tan DWM, Watt FM. Clonal Growth of Dermal Papilla Cells in Hydrogels Reveals Intrinsic Differences between Sox2-Positive and -Negative Cells In Vitro and In Vivo. Journal of Investigative Dermatology. 2012;132(4):1084–93. 10.1038/jid.2011.428 . 22189784 PMC3306894

[15] Morgan BA. The "skinny" on Wnt signaling in stem cells. Cell Stem Cell. 2013;13(6):638–40. 10.1016/j.stem.2013.11.012 . 24315435

[16] Zhang Y, Tomann P, Andl T, Gallant NM, Huelsken J, Jerchow B, et al Reciprocal requirements for EDA/EDAR/NF-kappaB and Wnt/beta-catenin signaling pathways in hair follicle induction. Dev Cell. 2009;17(1):49–61. Epub 2009/07/22. 10.1016/j.devcel.2009.05.011 19619491 PMC2859042

[17] Enshell-Seijffers D, Lindon C, Kashiwagi M, Morgan BA. beta-catenin activity in the dermal papilla regulates morphogenesis and regeneration of hair. Dev Cell. 2010;18(4):633–42. 10.1016/j.devcel.2010.01.016 20412777 PMC2893731

[18] Enshell-Seijffers D, Lindon C, Wu E, Taketo MM, Morgan BA. Beta-catenin activity in the dermal papilla of the hair follicle regulates pigment-type switching. Proc Natl Acad Sci U S A. 2010;107(50):21564–9. 10.1073/pnas.1007326107 21098273 PMC3003114

[19] Kaushal GS, Rognoni E, Lichtenberger BM, Driskell RR, Kretzschmar K, Hoste E, et al Fate of Prominin-1 Expressing Dermal Papilla Cells during Homeostasis, Wound Healing and Wnt Activation. J Invest Dermatol. 2015;135(12):2926–34. 10.1038/jid.2015.319 26288357 PMC4650270

[20] Muller-Rover S, Handjiski B, van der Veen C, Eichmuller S, Foitzik K, McKay IA, et al A comprehensive guide for the accurate classification of murine hair follicles in distinct hair cycle stages. J Invest Dermatol. 2001;117(1):3–15. 10.1046/j.0022-202x.2001.01377.x . 11442744

[21] Metzger D, Clifford J, Chiba H, Chambon P. Conditional Site-Specific Recombination in Mammalian-Cells Using a Ligand-Dependent Chimeric Cre Recombinase. P Natl Acad Sci USA. 1995;92(15):6991–5. 10.1073/pnas.92.15.6991 . PMC41457 7624356

[22] Madisen L, Zwingman TA, Sunkin SM, Oh SW, Zariwala HA, Gu H, et al A robust and high-throughput Cre reporting and characterization system for the whole mouse brain. Nat Neurosci. 2010;13(1):133–40. 10.1038/nn.2467 20023653 PMC2840225

[23] Iida M, Ihara S, Matsuzaki T. Hair cycle-dependent changes of alkaline phosphatase activity in the mesenchyme and epithelium in mouse vibrissal follicles. Dev Growth Differ. 2007;49(3):185–95. 10.1111/j.1440-169X.2007.00907.x . 17394597

[24] Belteki G, Haigh J, Kabacs N, Haigh K, Sison K, Costantini F, et al Conditional and inducible transgene expression in mice through the combinatorial use of Cre-mediated recombination and tetracycline induction. Nucleic Acids Res. 2005;33(5):e51 10.1093/nar/gni051 15784609 PMC1069131

[25] Kaufman CK, Zhou P, Pasolli HA, Rendl M, Bolotin D, Lim KC, et al GATA-3: an unexpected regulator of cell lineage determination in skin. Genes & development. 2003;17(17):2108–22. 10.1101/gad.1115203 12923059 PMC196453

[26] Lynch MH, Oguin WM, Hardy C, Mak L, Sun TT. Acidic and Basic Hair Nail (Hard) Keratins—Their Colocalization in Upper Cortical and Cuticle Cells of the Human-Hair Follicle and Their Relationship to Soft Keratins. Journal of Cell Biology. 1986;103(6):2593–606. 10.1083/jcb.103.6.2593 . 2432071 PMC2114622

[27] Kishimoto J, Ehama R, Wu L, Jiang S, Jiang N, Burgeson RE. Selective activation of the versican promoter by epithelial- mesenchymal interactions during hair follicle development. Proc Natl Acad Sci U S A. 1999;96(13):7336–41. 10377415 10.1073/pnas.96.13.7336 PMC22086

[28] Merrill BJ, Gat U, DasGupta R, Fuchs E. Tcf3 and Lef1 regulate lineage differentiation of multipotent stem cells in skin. Genes & development. 2001;15(13):1688–705. 10.1101/gad.891401 11445543 PMC312726

[29] Tetsu O, McCormick F. Beta-catenin regulates expression of cyclin D1 in colon carcinoma cells. Nature. 1999;398(6726):422–6. 10.1038/18884 . 10201372

[30] Collins CA, Jensen KB, MacRae EJ, Mansfield W, Watt FM. Polyclonal origin and hair induction ability of dermal papillae in neonatal and adult mouse back skin. Dev Biol. 2012;366(2):290–7. 10.1016/j.ydbio.2012.03.016 22537489 PMC3384004

[31] Zhang Y, Andl T, Yang SH, Teta M, Liu F, Seykora JT, et al Activation of beta-catenin signaling programs embryonic epidermis to hair follicle fate. Development. 2008;135(12):2161–72. Epub 2008/05/16. 10.1242/dev.017459 18480165 PMC2516408

[32] Vasioukhin V, Degenstein L, Wise B, Fuchs E. The magical touch: genome targeting in epidermal stem cells induced by tamoxifen application to mouse skin. Proc Natl Acad Sci U S A. 1999;96(15):8551–6. 10.1073/pnas.96.15.8551 10411913 PMC17554

