# Supplementary figures and images for: Activation of β-Catenin Signaling in CD133-Positive Dermal Papilla Cells Drives Postnatal Hair Growth
Source: PLoS One. 2016 Jul 29;11(7):e0160425. doi: 10.1371/journal.pone.0160425 (PMC4966972; doi:10.1371/journal.pone.0160425)

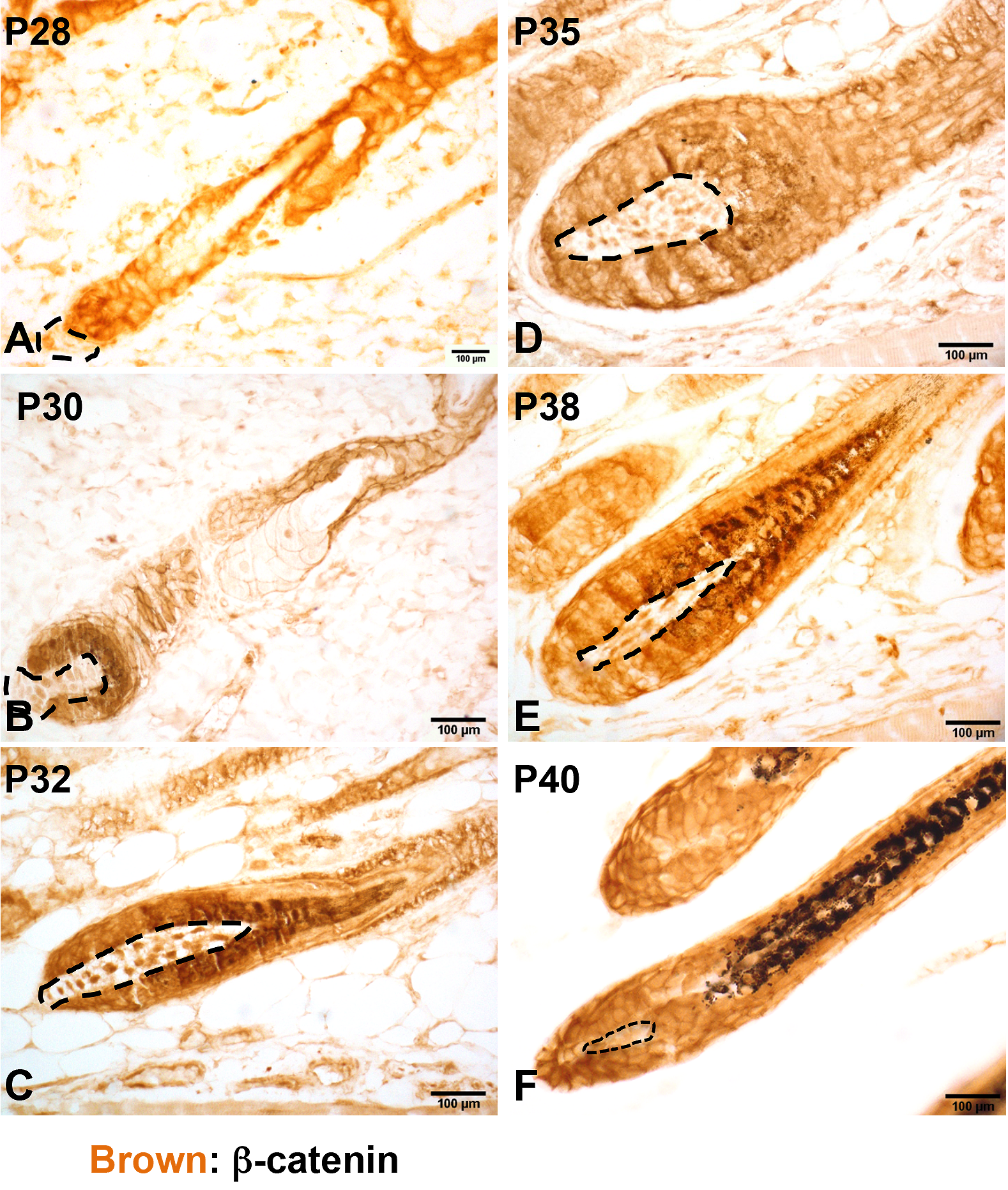

Supplement: S1 Fig — (A-F) Skin biopsies of normal C57BL/6 mice were collected at the indicated age and processed for paraffin sections. Expression of β-catenin (brown color) was visualized by immunohistochemistry. The DP was circled by white dashed lines in each hair follicle. Scale bar: 100 μm. (TIF) [file pone.0160425.s001.tif]

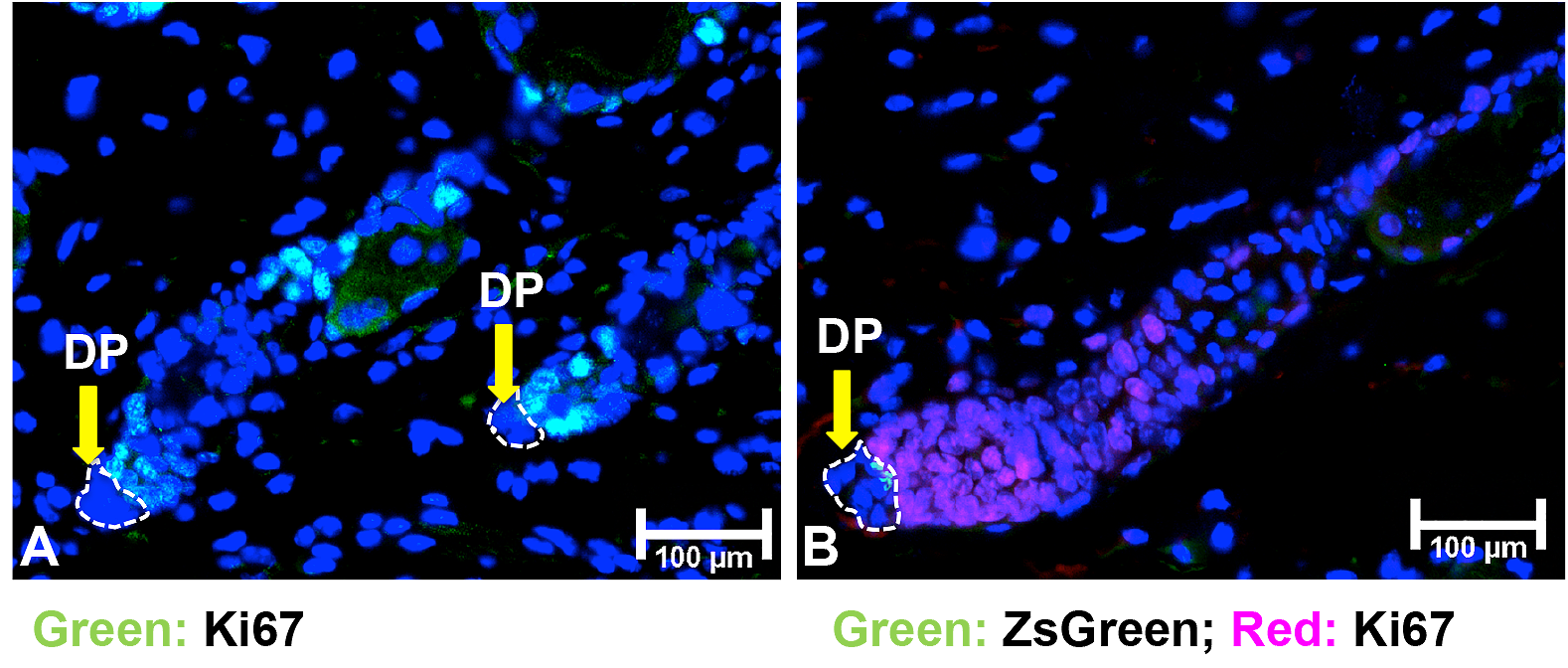

Supplement: S2 Fig — P28 skin biopsies of normal C57BL/6 mice (A) and CD133-CreERT2; ZsGreen1 mice (B) were analyzed for Ki67 expression by immunofluorescence staining. The DP was circled by white dashed lines in each hair follicle. Scale bar: 100 μm. (TIF) [file pone.0160425.s002.tif]

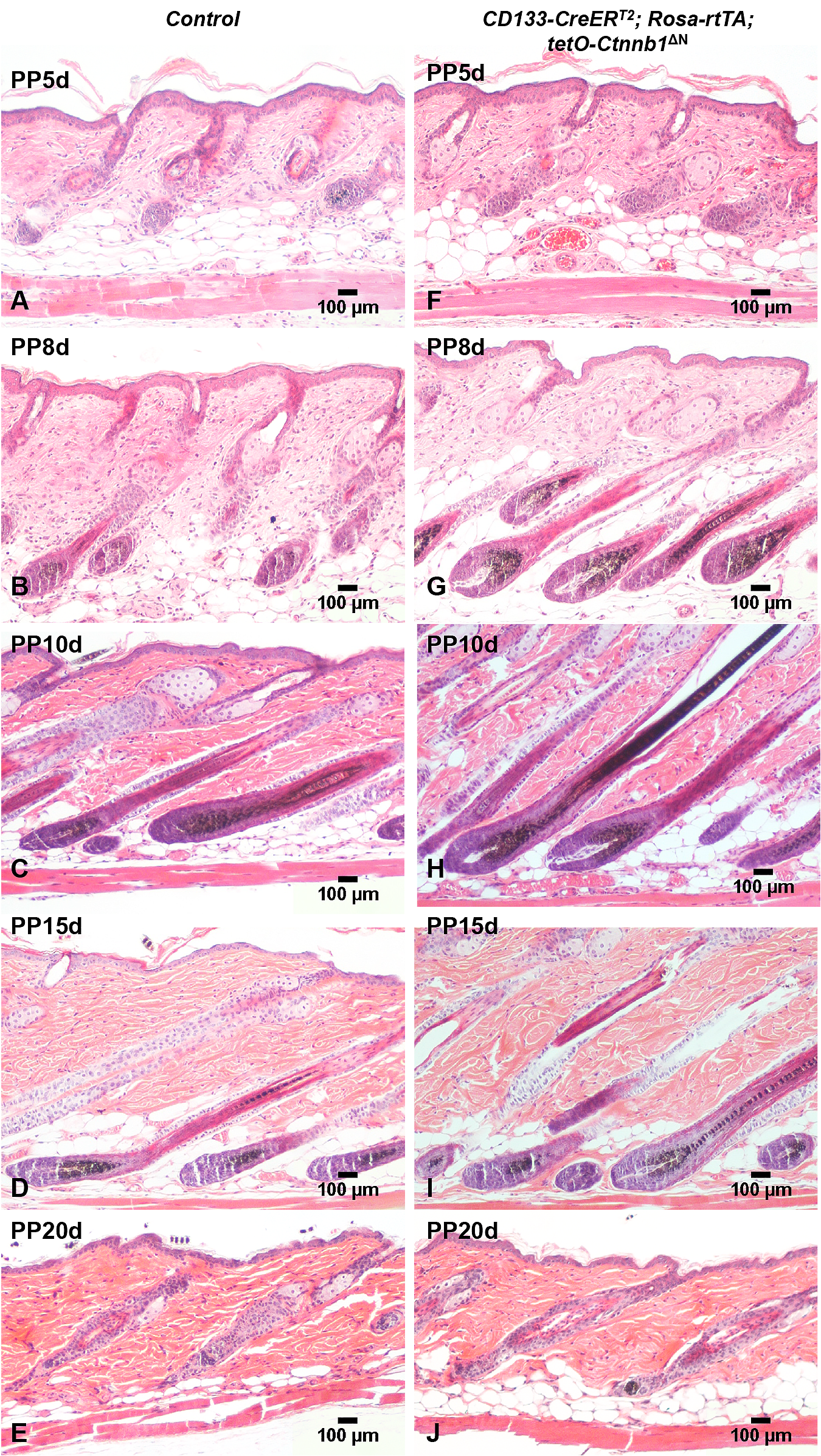

Supplement: S3 Fig — CD133-CreERT2; Rosa-rtTA; tetO-Ctnnb1ΔN mutant mice and control littermates were treated with tamoxifen from P50 for 7 days while on Dox diet, and their mid-dorsal hairs were plucked at each indicated age. Back skin biopsies from depilated areas of CD133-CreERT2; Rosa-rtTA; tetO-Ctnnb1ΔN mutant mice (F-J) and control littermates (A-E) were process for paraffin sections and stained with H&E. A minimum of three skin biopsies from three pairs of mutant and control mice was analyzed. Two-tailed paired Student’s t-test was employed to calculate statistical significance. Scale bars: 100 μm. (TIF) [file pone.0160425.s003.tif]

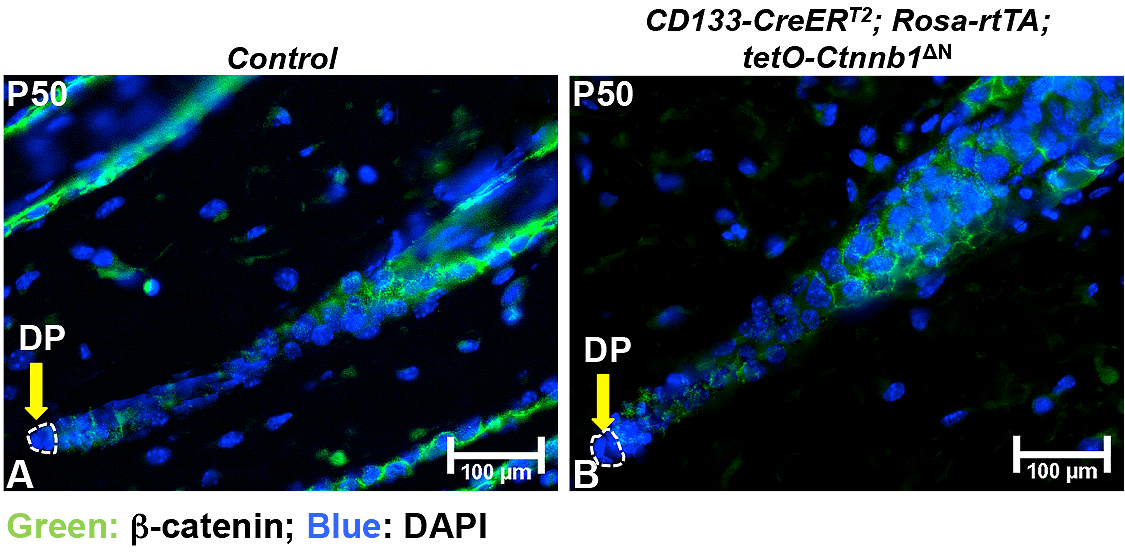

Supplement: S4 Fig — 5-μm-thick paraffin sections from P50 CD133-CreERT2; Rosa-rtTA; tetO-Ctnnb1ΔN mutant mice (B) and control littermates (A) were processed for β-catenin immunofluorescence staining. The DP was circled by white dashed lines in each hair follicle. Scale bar: 100 μm. (TIF) [file pone.0160425.s004.tif]
